# Gender equality and quality of life must be central to the design and delivery of sanitation

**DOI:** 10.1136/bmjgh-2024-018238

**Published:** 2025-01-22

**Authors:** Akanksha A Marphatia, Sheillah Simiyu, Meriel Flint O'Kane, Kelly T Alexander, Ana Carolina Argolo Nascimento de Castro, Ginette Azcona, Patience Esi Boni-Morkla, Salome A Bukachi, Phylis Busienei, Bethany A Caruso, Claire Chase, Jenala Chipungu, Anju Dwivedi, Richard Johnston, Indira Khurana, Antoinette Kome, Wanjiku Kuria, James Labadia, Fungai Makoni, Blessing Mberu, Sujoy Mojumdar, Janet Mule, Lydia Namatende Sakwa, Naomi Njeri, Fernanda Abreu Oliveira de Souza, Lauren Pandolfelli, Petunia Ramunenyiwa, Isha Ray, Malini Reddy, Pritum Kumar Saha, Utkarsh Sinha, Sheela S Sinharoy, Tom Slaymaker, Emmanuel Uguru, Kara Uhl, Sera L Young, Ian Ross, Oliver Cumming

**Affiliations:** 1 Department of Disease Control, London School of Hygiene and Tropical Medicine, London, UK; 2 Great Ormond Street Institute of Child Health, UCL, London, UK; 3 African Population and Health Research Center, Nairobi, Kenya; 4 CARE, Atlanta, Georgia, USA; 5 National Basic Water and Sanitation Agency, Federal Government of Brazil, Brasilia, Brazil; 6 Research and Data Section, UN Women, New York, New York, USA; 7 School of Public Policy and Global Affairs, University of British Columbia, Vancouver, BC, Canada; 8 Ministry of Sanitation and Water Resources, Government of Ghana, Accra, Ghana; 9 Department of Anthropology, Gender and African Studies, University of Nairobi, Nairobi, Kenya; 10 Hubert Department of Global Health, Rollins School of Public Health, Emory University, Atlanta, Georgia, USA; 11 Water Global Practice, World Bank Group, Washington, DC, USA; 12 Center for Infectious Disease Research in Zambia, Lusaka, Zambia; 13 Centre for Policy Research, New Delhi, India; 14 Department of Environment, Climate Change and Health, World Health Organization, Geneva, Switzerland; 15 Tarun Bharat Sangh, Bheekampura – Kishori, India; 16 SNV, Netherlands, The Hague, Netherlands; 17 World Vision Kenya, Nairobi, Kenya; 18 CARE South Sudan, Juba, Sudan; 19 Water Sanitation and Hygiene Team, UNICEF India, New Delhi, India; 20 Department of Environmental Health and Sanitation, Government of Kenya, Nairobi, Kenya; 21 Division of Data, Analytics, Planning and Monitoring, UNICEF, New York, New York, USA; 22 Department of Water and Sanitation, Government of South Africa, Pretoria, South Africa; 23 Energy and Resources Group, UC Berkeley, Berkeley, California, USA; 24 Re Sustainability Limited, Hyderabad, India; 25 Water and Sanitation for the Urban Poor, Dhaka, Bangladesh; 26 Parmarth Samaj Sevi Sansthan, Jalaun, India; 27 African Ministers Council on Water, Abuja, Nigeria; 28 Bill and Melinda Gates Foundation, Seattle, Washington, USA; 29 Department of Anthropology, Northwestern University, Evanston, Illinois, USA

**Keywords:** Global Health, Decision Making, Environmental health, Health policy, Interdisciplinary Research

**Summary box**

- Sanitation is critical to improving quality of life and gender equality.
- How we understand and—value sanitation—requires critical reflection and greater consideration of the different experiences of women, men, girls and boys.
- Sanitation interventions can cause harm to women and girls, and these risks must be mitigated through a do-no-harm approach.
- Harnessing the full benefits of sanitation, beyond the reduction of disease, and including quality of life and gender equality requires intentional design and investment.
- Incorporating both quality of life and gender equality into the monitoring and evaluation of sanitation services is important, and mutually reinforcing.
- Women’s leadership across the sanitation sector is central to achieving this agenda.

## Introduction

 In 2015, 191 United Nations (UN) member states agreed to a new Sustainable Development Goal (SDG) for Water, Sanitation and Hygiene (WASH), setting a commitment to, ‘By 2030, achieve access to adequate and equitable sanitation… paying special attention to the needs of women and girls…’.^1^ And, the human right to sanitation asserts the importance of sanitation for quality of life, including safety, security, privacy and dignity.^2^ However, gender equality and quality of life are rarely central to how sanitation is understood, delivered, measured and evaluated. Furthermore, the adverse effects of climate change on access to sanitation services will negatively impact on quality of life and disproportionately affect women.^3^

In the drinking water sector, research shows that women often experience water insecurity differently from men,^4^ but there is less evidence on how women and men experience sanitation insecurity. Under the capability approach to quality of life, a good life calls for people to be free to do the things in life they value.^5^ Sanitation-related quality of life refers to how sanitation practices and services directly affect people’s lived experiences (eg, privacy and safety).^6^ Gender equality means addressing gaps in freedoms, opportunities and outcomes among women, men, girls, boys and people of diverse identities, arising from both de jure (legal) and de facto (actual) inequalities.^7^ This definition of gender equality acknowledges systemic barriers that obstruct equal rights and emphasises the need for measures that promote formal and substantive equality, enabling all individuals to realise their full potential, rights and dignity.^7^

## Recommendations

In February 2024, the African Population Health Research Center and the London School of Hygiene and Tropical Medicine convened a meeting in Nairobi, Kenya, to discuss the linkages between sanitation, gender equality and quality of life, and how to enhance the sector’s contribution to these outcomes. Approximately 50 participants participated in the meeting, including government representatives, international agencies, operational organisations and academic research institutions from different regions of the world. Points of consensus emerged which we share here to stimulate broader reflection and discussion in the sanitation sector and beyond.

## How we understand sanitation and its importance to individuals and society requires critical reflection

Sanitation is generally defined as the separation of human waste from human contact for the purpose of preventing disease transmission,^8^ and the sanitation attributable burden of infectious disease remains important.^9^ This definition, however, does not account for the full impact of inadequate sanitation on individuals and society and fails to recognise the importance of menstrual hygiene and health. Drawing on the World Health Organization’s (WHO) definition of health, we argue for the full impact of inadequate sanitation on individuals and society to be recognised.^10^ This includes the contribution of sanitation to quality of life, human dignity, well-being, the environment, etc, and, ‘not only the absence of disease’. Therefore, how we understand and measure the impact of sanitation must reflect how the diverse experiences of women, men, girls, boys and other identities intersect with other social characteristics, such as age, ethnicity, religion, class and disability, to impact their quality of life.^11^

## At a minimum, sanitation interventions should do no harm

Preventing the transmission of infectious diseases is critical but not at the expense of quality of life and gender equality. A recent re-review of WASH interventions^12^ which used the Gender Responsive Assessment Scale (GRAS),^13^ classified the included sanitation interventions as either gender unequal or gender unaware, indicating an exploitative engagement with women.^12^ Yet, current approaches often fail to recognise and respond to women’s needs.^14^ We urge all sanitation stakeholders to use the GRAS or other tools to explicitly measure the impact of sanitation interventions on gender equality and quality of life. All organisations should develop and monitor do-no-harm strategies addressing gender-specific (eg, safety) and sex-specific (eg, menstruation) needs, with the aim of transforming biased gendered norms, dynamics and structures.

## Potential gains in quality of life and gender equality cannot be realised without intentional design and investment

Women, men, girls and boys and other identities have an equal right to expect access to affordable and sustainable sanitation services that enhance their quality of life.^2^ Policies, plans, financing and regulations should prioritise and protect gender-equitable access to sanitation at home, in public spaces and institutions. Stakeholders must go beyond box-ticking (eg, counting the number of toilets for women and men) by setting up structures and accountability mechanisms that ensure sustained progress on gender equality^15^ and sanitation-related quality of life. Both the benefits of investing in gender equality and quality of life, and the consequences of not doing so, should be estimated. All this requires assessing and strengthening education, training, research and sustained commitment from relevant staff at all levels, and recognition that progress on gender equality begins within our own organisations.^11 16^

## Including indicators of gender equality and quality of life in the monitoring of sanitation services will increase awareness and accountability of actors

Recent research has produced new indicators, scales and indices to monitor and measure gender equality, women’s empowerment and quality of life in relation to sanitation at policy, operational and research levels. The WHO/UNICEF Joint Monitoring Programme for Water Supply, Sanitation and Hygiene has three gender-specific indicators measuring differential access and experience of sanitation in relation to cleanliness, privacy and safety.^17^ The following are just a few examples of the many tools that can underpin the collection of comparable sex-disaggregated quality of life data across and within countries—the Sanitation-related Quality of Life (SanQoL-5) index measures gaps in privacy, safety, cleanliness and other outcomes between women and men^6^; the WASH-Gender Equality Measure (WASH-GEM) assesses changes in gender dynamics at the programmatic level^18^; and Safety Audits record community perceptions and experience of sanitation infrastructure and services.^19^ In addition, the Agency, Resources, and Institutional Structures for Sanitation-related Empowerment (ARISE) scales is an example of a gender-specific measure that can be used with women to measure various subdomains of empowerment (eg, decision-making, safety norms).^20^ Greater efforts are also required to understand intersectionality, or how other aspects such as age, ethnicity, class, disability and religion intersect with gender and quality of life to compound inequality. The research community has an important role to play in generating this evidence. Stronger implementation capacity, meaningful participation of local stakeholders and the inclusion of women as change leaders in communities and in formal and informal leadership positions in these efforts are long overdue.^11^

## The under-representation of women in leadership positions in the sanitation sector must be addressed urgently

Women play a key role in delivering sanitation interventions but continue to be left out of decision-making.^12^ Achieving sanitation-related gender equality and quality of life outcomes requires strong leadership and accountability on these issues at all levels. Policy, operational and research institutions should urgently address the under-representation of women in leadership positions.^16^ Education and training should be provided across sanitation and related sectors (eg, water) so women can access and be influential in decision-making positions.^11^ This includes awareness and training for men to advocate for and support women leaders in informal and formal sanitation initiatives and institutions.

## Conclusion

As we pass the mid-point of the SDG period (2015–2030) and look ahead to new goals and targets, we argue that gender equality and quality of life must be central to how sanitation is understood, measured, designed and delivered. Achieving gains in these inter-related areas requires the intentional design of sanitation policies, plans and services, financing the meaningful promotion of women leaders, engagement of local stakeholders and rights-holders, and commitment to the broader goal of gender transformation in related sectors. To ensure progress on gender equality and quality of life, sustained monitoring is required to make sure efforts are on track and to enable course correction as needed.

## Data Availability

There are no data in this work.

## References

[1] United Nations General Assembly (2015). Transforming our world: The 2030 agenda for Sustainable Development. http://sdgs.un.org/2030agenda.

[2] United Nations General Assembly (2010). The human right to water and sanitation. https://documents.un.org/doc/undoc/gen/n09/479/35/pdf/n0947935.pdf.

[3] van Daalen KR, Jung L, Dada S (2024). Bridging the gender, climate, and health gap: the road to COP29. Lancet Planet Health.

[4] Young SL, Bethancourt HJ, Ritter ZR (2022). Estimating national, demographic, and socioeconomic disparities in water insecurity experiences in low-income and middle-income countries in 2020–21: a cross-sectional, observational study using nationally representative survey data. Lancet Planet Health.

[5] Sen A, Nussbaum M, Sen A (1993). The Quality of Life.

[6] Akter F, Banze N, Capitine I The Sanitation-related Quality of Life index (SanQol-5) – validity and reliability in rural and urban settings in Ethiopia, Malawi, Mozambique, and Zambia. In Review.

[7] Committee on the Elimination of Discrimination Against Women (2004). General Recommendation No. 25 on Article 4, Paragraph 1 of the Convention on the Elimination of All Forms of Discrimination Against Women. http://www.un.org/womenwatch/daw/cedaw/recommendations/General%20recommendation%2025%20(English).pdf.

[8] World Health Organization (2018). Guidelines on sanitation and health. http://iris.who.int/bitstream/handle/10665/274939/9789241514705-eng.pdf?sequence=25.

[9] Wolf J, Johnston RB, Ambelu A (2023). Burden of disease attributable to unsafe drinking water, sanitation, and hygiene in domestic settings: a global analysis for selected adverse health outcomes. The Lancet.

[10] World Health Organization (1948). Constitution of the World Health Organization. http://www.who.int/about/governance/constitution.

[11] UN Women, UN-Water (2023). Spotlight on SDG 6: from commodity to common good: A feminist agenda to tackle the world’s water crisis. http://www.unwomen.org/en/digital-library/publications/2023/07/from-commodity-to-common-good-a-feminist-agenda-to-tackle-the-worlds-water-crisis.

[12] Caruso BA, Ballard AM, Sobolik J (2024). Systematic re-review of WASH trials to assess women’s engagement in intervention delivery and research activities. *Nat Water*.

[13] WHO Gender Equity Unit (2023). Introduction to gender responsive assessment and gender levels. https://genderhealthdata.org/resource/introduction-to-gender-responsive-assessment-and-gender-levels/.

[14] MacArthur J, Carrard N, Willetts J (2020). WASH and Gender: a critical review of the literature and implications for gender-transformative WASH research. J Water Sanit Hyg Dev.

[15] Tilley E, Bieri S, Kohler P (2013). Sanitation in developing countries: a review through a gender lens. J Water Sanit Hyg Dev.

[16] Oluwasanya G, Omoniyi A, Qadir M (2024). Quantifying women in the water workforce. *Nat Water*.

[17] Caruso BA, Chipungu J, Hennegan J (2024). Priority gender-specific indicators for WASH monitoring under SDG targets 6.1 and 6.2: Recommendations for national and global monitoring. http://washdata.org/reports/emory-2024-priority-gender-specific-indicators-for-wash-monitoring.

[18] MacArthur J, Chase RP, Gonzalez D (2024). Investigating impacts of gender-transformative interventions in water, sanitation, and hygiene: Structural validity, internal reliability and measurement invariance of the water, sanitation, and hygiene–Gender equality measure (WASH-GEM). *PLOS Water*.

[19] Labadia J, Alexander K (2024). WASH safety audits tool: Water point safety and access to an existing/new water point. http://www.care.org/resources/safetyauditwash.

[20] Sinharoy SS, McManus S, Conrad A (2023). The Agency, Resources, and Institutional Structures for Sanitation-related Empowerment (ARISE) Scales: Development and validation of measures of women’s empowerment in urban sanitation for low- and middle-income countries. World Dev.

